# First tarsometatarsal joint mobility in hallux valgus during gait: A synchronized ultrasound and three-dimensional motion capture analysis

**DOI:** 10.1007/s10396-024-01414-2

**Published:** 2024-03-28

**Authors:** Tsubasa Tashiro, Yasunari Ikuta, Noriaki Maeda, Satoshi Arima, Masanori Morikawa, Kazuki Kaneda, Honoka Ishihara, Shogo Tsutsumi, Miki Kawai, Andreas Brand, Tomoyuki Nakasa, Nobuo Adachi, Makoto Komiya, Yukio Urabe

**Affiliations:** 1 Department of Sports Rehabilitation, Graduate School of Biomedical and Health Sciences, Hiroshima University, 1-2-3 Kasumi, Minami-Ku, Hiroshima, 734-8553 Japan; 2 Department of Orthopedic Surgery, Graduate School of Biomedical and Health Sciences, Hiroshima University, Hiroshima, 734-8551 Japan; 3 Sports Medical Center, Hiroshima University Hospital, Hiroshima, 734-8551 Japan; 4 Department of Preventive Gerontology, Center for Gerontology and Social Science, National Center for Geriatrics and Gerontology, Aichi, 474-8511 Japan; 5 Institute for Biomechanics, BG Unfallklinik Murnau, Murnau, Germany; 6 Institute for Biomechanics, Paracelsus Medical Private University Salzburg, Salzburg, Austria; 7 Medical Center for Translational and Clinical Research, Hiroshima University Hospital, Hiroshima, 734-8551 Japan

**Keywords:** First tarsometatarsal joint, Hallux valgus, Gait, Ultrasound

## Abstract

**Purpose:**

To quantify the vertical translation between the first metatarsal and medial cuneiform during the stance phase of gait in young individuals with and without hallux valgus.

**Design:**

This cross-sectional observational study included 34 young adults (male, *n* = 4; female, *n* = 30) who were divided into three groups according to the hallux valgus angle: control (< 20°, *n* = 13), mild hallux valgus (≥ 20° to < 30°, *n* = 12), and moderate hallux valgus (≥ 30°, *n* = 9). The mobility of the first tarsometatarsal joint was evaluated during the stance phase using B-mode ultrasound synchronized with a motion analysis system.

**Results:**

The medial cuneiform shifted more plantar during the early phase in mild hallux valgus and during the middle and terminal phases in moderate hallux valgus than in control. The severity of the hallux valgus was correlated with a trend toward plantar shift of the medial cuneiform. The first metatarsal was located more dorsal than the medial cuneiform; however, there was no significant variation. No significant differences in the peak ankle plantarflexion angle and moment were noted between the groups.

**Conclusion:**

The hypermobility of the first tarsometatarsal joint, especially plantar displacement of the medial cuneiform in the sagittal plane, was found in young individuals with hallux valgus during the stance phase of gait, and the mobility increased with the severity of hallux valgus. Our findings suggest the significance of preventing hallux valgus deformity early in life.

**Supplementary Information:**

The online version contains supplementary material available at 10.1007/s10396-024-01414-2.

## Introduction

Hallux valgus (HV) is a common foot deformity that impairs the health-related quality of life as its severity increases [1]. The severity of HV highly correlates with hypermobility of the first ray, which is a causative factor for HV [2, 3]. The first tarsometatarsal (TMT) joint exhibited hypermobility in up to 96% of patients with recurrent HV [4, 5]. Thus, the preoperative first TMT joint hypermobility should be evaluated accurately. Most previous studies focused on static evaluations, including manual manipulation of the first metatarsal, the Klaue device, and stress radiographs [5–7]. These methods can be easily applied in clinical practice; however, the validity and reliability are particular concerns because of the manual intervention and low inter-rater reliability [6].

Three-dimensional (3D) motion is produced in the medial column of the foot during weight-bearing [8], and the main components of the first metatarsal motion occur in the sagittal plane [9]. Repetitive loading during gait can lead to plantar ligament laxity of the first TMT joint, which reduces foot stiffness during the stance phase and disrupts push-off mechanics [9]. Subsequently, greater dorsiflexion of the first metatarsal promotes the development of HV [6, 9, 10]. Accordingly, the hypermobility of the first TMT joint should be evaluated dynamically during gait. Recently, dynamic evaluation methods have been widely used in gait analysis. Biomechanical studies using a 3D motion analysis (MA) system described the angle of the first TMT joint during gait [11]. However, this method has limitations in terms of a detailed assessment of joint mobility due to skin artifacts [12] and small joint movements in the foot. Moreover, no significant association was observed between the instability of the first TMT joint on static weight-bearing lateral radiographs and dynamic pedobarographic findings, which was associated with a greater HV angle (HVA) [13].

Therefore, we developed a novel combination of synchronized ultrasound (US) imaging and a 3D-MA (US/MA) system for quantitative evaluation of first TMT joint dynamics during gait [14]. Quantitative analysis of first TMT joint mobility under dynamic conditions can potentially aid in identification of the pathogenesis of HV and prevention of recurrence of HV through appropriate treatment. Previous fluorokinematic analysis predicted that the severity of HV influences the dynamics of the first TMT joint [2]. Therefore, we hypothesized that first TMT joint mobility during gait is associated with HV severity. The present study aimed to quantify the vertical translation between the first metatarsal and medial cuneiform during the stance phase of gait, and compare the mobility among young individuals with and without HV using a novel combination of the US/MA system.

## Materials and methods

### Study design and participants

This cross-sectional observational study was performed in accordance with the Strengthening the Reporting of Observational Studies in Epidemiology (STROBE) guidelines. Data were collected between October 2020 and May 2021. Thirty-four participants were included in the study (Table 1). The exclusion criteria were a score ≥ 6 on the Foot Posture Index [15]; ligament injuries, plantar fasciitis, bursitis, or orthopedic injuries to the hip, knee, ankle, or foot; a history of lower extremity trauma; and neurological conditions that can affect gait.

**Table 1:** Summary of the participants’ data

	Control (*n* = 13)	Mild HV (*n* = 12)	Moderate HV (*n* = 9)	*F*	*P*-value^a^	*P*^b^	*P*^c^	*P*^d^
Sex, female	11 (84.6)	11 (91.7)	8 (88.9)					
Age (years)	23.7 ± 4.7	21.2 ± 1.3	21.3 ± 1.5	2.502	0.360			
Height (cm)	158.3 ± 5.1	161.1 ± 5.5	160.3 ± 5.9	0.885	0.423			
Body weight (kg)	51.7 ± 6.7	51.7 ± 4.2	53.8 ± 6.8	0.417	0.663			
Body mass index (kg/m^2^)	20.6 ± 1.9	19.9 ± 1.0	20.9 ± 1.5	1.139	0.333			
Hallux valgus angle (°)	8.7 ± 5.0	23.2 ± 1.5	33.9 ± 3.3	96.782	< 0.001	0.005	< 0.001	0.050
Foot circumference (cm)	22.0 ± 1.4	22.2 ± 1.0	22.5 ± 0.9	0.381	0.686			
Foot Posture Index	2.5 ± 1.6	2.6 ± 1.7	3.3 ± 1.8	0.694	0.458			
SAFE-Q subscale								
Pain and pain-related (100)	98.9 ± 4.0	89.6 ± 11.5	83.6 ± 15.0	7.126	0.008	0.059	0.011	1.000
Physical functioning and daily living (100)	98.6 ± 3.8	98.7 ± 1.8	99.2 ± 1.6	0.168	0.558			
Social functioning (100)	100.0 ± 0.0	98.6 ± 3.7	99.5 ± 1.4	1.150	0.332			
Shoe-related (100)	98.1 ± 3.7	86.8 ± 13.0	76.9 ± 14.9	11.781	0.001	0.090	0.001	0.390
General health and well-being (100)	100.0 ± 0.0	97.5 ± 5.0	98.9 ± 3.3	1.663	0.162			

Values are presented as *n* (%) or mean ± standard deviation

HV, hallux valgus; SAFE-Q, Self-Administered Foot Evaluation Questionnaire

^a^Results of one-way analysis of variance or Kruskal–Wallis test and of multiple comparisons according to Bonferroni’s correction method ^b^between control and mild HV, ^c^between control and moderate HV, and ^d^between mild and moderate HV

To categorize participants according to the severity of the HV deformity, the HVA was measured from a photograph of the participant’s foot taken during weight-bearing. The photo was taken from the horizontal plane at knee height by an examiner using an iPhone X (Apple, Cupertino, CA, USA) [16]. The HVA was defined as the angle between the medial aspect of the hallux and the first metatarsal. Using these measurements, participants were classified into three groups: control (HVA < 20°, *n* = 13), mild HV (HVA ≥ 20° to < 30°, *n* = 12), and moderate HV (HVA ≥ 30° to < 40°, *n* = 9) [17]. In the mild and moderate HV groups, the foot with the greater HVA was selected for the subsequent analyses [18], whereas both feet were used in the control. Ultimately, 26 feet in the control, 12 feet in the mild HV, and nine feet in the moderate HV groups were selected for analysis. The foot circumference was measured transversely using a tape measure at the level of the first TMT joint. The average value of three measurements was calculated as the foot circumference. Additionally, participants completed the Self-Administered Foot Evaluation Questionnaire (SAFE-Q) [19]. The SAFE-Q comprises 34 questions with five subscale scores related to “pain and pain-related,” “physical functioning and daily living,” “social functioning,” “shoe-related,” and “general health and well-being.”

The study protocols complied with the principles laid down in the Declaration of Helsinki and were approved by the Ethical Committee for Epidemiology of Hiroshima University (Approval number: E-2187). All participants provided informed consent for participation in the study.

### Experimental procedures

#### Assessment of first TMT joint mobility using the B-mode US system during gait

A B-mode US system (Art Us EXT-1H; Telemed, Vilnius, Lithuania) with a US probe (5–11 MHz, 60-mm field of view; Echo Blaster, Telemed) was used to evaluate the motion of the first TMT joint in the sagittal plane during the stance phase of gait [20]. The setting of the US probe is shown in Supplementary Fig. 1. The US probe was positioned longitudinally over the first TMT joint. A US gel pad (Yasojima Proceed Co., Ltd., Kobe, Japan) was placed between the dorsum of the foot and the US probe to improve image quality and avoid compression of the skin surface. The US probe was adjusted to a position where the first TMT joint was visible on the screen and then fixed in position with an elastic band (Supplementary Fig. 1). The US system was synchronized with a 3D motion capture analysis system (VICON MX T20-S; Vicon Motion Systems, Oxford, UK), and the start of the MA system triggered the simultaneous capture of a B-mode US video at a frame rate of 80 frames/s and an image depth of 60 mm. This synchronization system was standardized based on the established technique [14]. Three trials of US video recordings were measured for each side. The position of the probe was checked after each trial and modified if necessary. Trained physiotherapists with at least 3 years of experience in US imaging evaluated the US measurements.

#### Gait analysis

Participants walked along a 6-m walking path with eight force plates (OR-6, 1000 Hz; AMTI, Watertown, MA, USA). Gait analysis was performed using the MA system with 16 infrared cameras at a 100-Hz sampling frequency (Bonita; Vicon, Hauppauge, NY, USA) [21]. First, 16 reflective markers (14 mm in diameter) were placed on the right and left sides of the anterior superior iliac spine, posterior superior iliac spine, thigh, lateral knee joint, lateral lower leg, lateral malleolus, calcaneal tuberosity, and second metatarsal head by the same examiner [22]. The static posture was measured with the participants in the anatomical standing position. Then, the participants walked over the force plates at a comfortable speed, looking ahead. After two practice sessions, three gait trials were performed. Both feet were recorded and measured in the control group.

#### Data analysis

The gait parameters of the probe-wearing foot, including the cadence, walking speed, stride time, step time, stride length, and step length, were calculated. The force plate data were processed using the plug-in-gait pipeline for Vicon Nexus version 1.8.5 (Vicon Motion Systems) to detect the gait phase. The phase from heel contact to toe-off (the complete stance phase) of the foot was identified and analyzed with the US probe. One stance phase was calculated for each of the three trials on each side. Tracker 5.1.5 (Open Source Physics, https://www.compadre.org) was used to calculate the vertical translation of the metatarsal and medial cuneiform in the US video [23]. The temporal changes in the vertical location of the first TMT joint during the stance phase of gait obtained using the Vicon software were calculated. The vertical locations of the first metatarsal and medial cuneiform were defined as the perpendicular distance from the top of the US video screen to that of each bone (Supplementary Fig. 2), and their locations were calculated for all frames. The gap in the vertical location of the medial cuneiform relative to the first metatarsal was analyzed by defining the first frame as the zero level. Normalization software was used to normalize one stance phase for each participant to 100 frames. Finally, each stride between heel contact and toe-off was analyzed separately for the early stance phase (0–33 frames), middle stance phase (34–66 frames), and terminal stance phase (67–100 frames) [24]. All videos were analyzed by examiners who were blinded to the participant’s information.

The ankle dorsiflexion and plantarflexion angle and moment during one gait cycle were also calculated. These data were obtained from the foot of the probe-wearing side. The ankle moments were normalized by body weight.

#### Statistical analysis

All statistical analyses were performed using SPSS for Windows version 23.0 (IBM Corp., Armonk, NY, USA). The data were tested for data distribution normality using the Shapiro–Wilk test. For items including participant demographic data, gait parameters, peak ankle plantarflexion angle, and peak ankle plantarflexion moment, a one-way analysis of variance (ANOVA) was performed if normality existed, and the Kruskal–Wallis test was performed if not. Differences in US evaluation values for all outcome measures were determined using a two-way repeated-measures ANOVA with any group (control, mild HV, and moderate HV) as the between-participant factor and time (early, middle, and terminal stance) as the within-participant factor. Post-hoc comparisons using Bonferroni's correction method were performed when interaction effects were found to test for differences in the first metatarsal and medial cuneiform motion variables among the three groups. Partial η^2^ values were reported as measures of effect size. The post-hoc observed power based on partial η^2^ was generated using G*Power 3.1 (Kiel University, Kiel, Germany). Additionally, the Jonckheere–Terpstra test was used to examine the trend in the vertical location of the first TMT joint during the stance phase of gait across the three groups: control, mild HV, and moderate HV. The intra-rater reliability of vertical movement of the first TMT joint during the three stance phases was assessed with intraclass correlation coefficients (ICC1,3). ICC1,3 was regarded as excellent if > 0.74, good if between 0.60 and 0.74, fair if between 0.40 and 0.59, and poor if < 0.40. The standard error of the measurements was also determined to confirm the accuracy of the measurements. The statistical significance level (*P*) was set at < 0.05.

The required sample size was calculated with a post-hoc power analysis using a partial η^2^ of 0.257 for the within-group main effect of the medial cuneiform. The post-hoc power calculation based on an *F* test and repeated measures within-between interaction ANOVA, which had a large effect size (*f* = 0.59) and alpha level (*P* < 0.05), showed a statistical power of 0.819 and a total sample size of 33. Therefore, there was adequate power and sample size at the large effect size level.

## Results

The participant demographics of the control (*n* = 13), mild HV (*n* = 12), and moderate HV (*n* = 9) groups are shown in Table 1. Significant differences were found in the HVA between the control and mild HV groups (*P* = 0.005) and between the control and moderate HV groups (*P* < 0.001). Several SAFE-Q subscales (pain and pain-related, shoe-related) were significantly different between the control and moderate groups (*P* = 0.011 and *P* = 0.001, respectively). No significant differences were found in gait parameters among each group (Table 2).

**Table 2:** Gait parameters

	Control	Mild HV	Moderate HV	*F*	*P*-value
Cadence (steps/min)	102.92 ± 6.46	102.56 ± 7.29	96.82 ± 7.00	2.491	0.096
Walking speed (m/s)	1.00 ± 0.08	0.98 ± 0.07	0.93 ± 0.08	2.086	0.138
Stride time (s)	1.17 ± 0.08	1.18 ± 0.09	1.28 ± 0.15	3.197	0.052
Step time (s)	0.60 ± 0.05	0.60 ± 0.05	0.68 ± 0.16	2.927	0.066
Stride length (m)	1.16 ± 0.04	1.15 ± 0.04	1.15 ± 0.05	0.373	0.691
Step length (m)	0.60 ± 0.02	0.57 ± 0.05	0.59 ± 0.03	2.078	0.139

Values are presented as mean ± standard deviation

Results of one-way analysis of variance

HV, hallux valgus

**Fig. 1:**
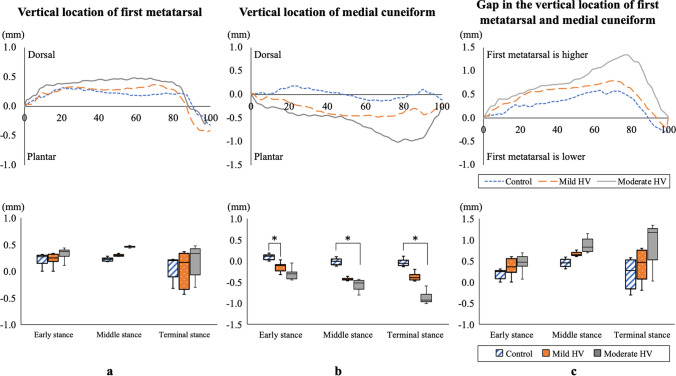
Temporal changes in the vertical location of the first metatarsal (**a**), vertical location of the medial cuneiform (**b**), and displacement of the first metatarsal relative to the medial cuneiform (**c**) during the stance phase of gait are shown for control, mild HV, and moderate HV. Box plots comparing the vertical location of the first metatarsal, vertical location of the medial cuneiform, and relative location of the two during the early, middle, and terminal stance phases are shown for control, mild HV, and moderate HV. *P < 0.05. HV, hallux valgus

The temporal kinematic parameters of the first TMT joint during the stance phase of the gait cycle are shown in Fig. 1. No significant differences were found in the temporal change of the first metatarsal among the three groups (Fig. 1a). Compared with the control group, the medial cuneiform was located more plantar in the mild HV group during the early gait stance phase (*P* = 0.013), and in the moderate HV group during the middle and terminal gait stance phases (*P* = 0.042 and *P* = 0.002, respectively) (Fig. 1b). The gap in the vertical location of the first metatarsal and medial cuneiform tended to be large throughout the stance phase according to HV severity. However, no statistical differences were identified (Fig. 1c).

Table 3 presents the results of the two-factor (group × phase) ANOVA with repeated measures and the Jonckheere–Terpstra test. The group significantly affected the vertical locations of the medial cuneiform during the stance phase (*P* = 0.001). Time, including early, middle, and terminal stance phases, had a significant effect on the vertical locations of the first metatarsal and medial cuneiform and the gap between them (*P* < 0.001, *P* < 0.001, and *P* = 0.002, respectively). The Jonckheere–Terpstra trend test results showed that only plantar displacement of the medial cuneiform increased between control, mild HV, and moderate HV across all gait stance phases (early stance phase: standardized statistic [SS] = -3.135, *P* = 0.002; middle stance phase: SS = -2.848, *P* = 0.004; terminal stance phase: SS = − 3.217, *P* = 0.001).

**Table 3:** Displacement of the first metatarsal, medial cuneiform, and gap during the stance phase of gait

	Control	Mild HV	Moderate HV	Interaction effect (group × time)					Main effect (group)					Main effect (time)					Post-hoc			^d^SS	^d^*P* for Trend	
				*F*	*P*	η^2^		Observed power	*F*	*P*	η^2^		Observed power	*F*	*P*	η^2^		Observed power	*P*^a^	*P*^b^	*P*^c^			
First metatarsal (mm)																								
Early stance phase	0.22 ± 0.28	0.23 ± 0.21	0.32 ± 0.50	0.51	0.728	0.023	0.167		0.417	0.661	0.019	0.113		9.117	< 0.001	0.172	0.972		1.000	1.000	1.000	1.332		0.183
Middle stance phase	0.22 ± 0.46	0.30 ± 0.44	0.46 ± 0.88																1.000	0.828	1.000	1.721		0.085
Terminal stance phase	0.08 ± 0.41	0.04 ± 0.51	0.18 ± 0.70																1.000	1.000	1.000	0.492		0.623
Medial cuneiform (mm)																								
Early stance phase	0.09 ± 0.24	− 0.15 ± 0.21	− 0.32 ± 0.51	1.616	0.177	0.068	0.479		7.609	0.001	0.257	0.931		9.157	< 0.001	0.172	0.972		0.013	0.100	0.620	− 3.135		0.002
Middle stance phase	− 0.02 ± 0.50	− 0.43 ± 0.45	− 0.56 ± 0.78																0.111	0.042	1.000	− 2.848		0.004
Terminal stance phase	− 0.04 ± 0.48	− 0.38 ± 0.49	− 0.83 ± 0.79																0.254	0.002	0.202	− 3.217		0.001
Gap in the first metatarsal and medial cuneiform (mm)																								
Early stance phase	− 0.19 ± 0.51	− 0.36 ± 0.25	− 0.45 ± 0.63	1.595	0.201	0.068	0.393		1.433	0.249	0.061	0.291		8.718	0.002	0.165	0.913		0.913	0.505	1.000	− 1.742		0.082
Middle stance phase	− 0.45 ± 0.90	− 0.66 ± 0.53	− 0.86 ± 0.86																1.000	0.622	1.000	− 0.983		0.325
Terminal stance phase	− 0.20 ± 1.09	− 0.39 ± 0.45	− 0.91 ± 0.79																1.000	0.161	0.628	− 1.844		0.065

Values are presented as mean ± standard deviation

Results of multiple comparisons according to Bonferroni’s correction method ^a^between control and mild HV, ^b^between control and moderate HV, and ^c^between mild HV and moderate HV, ^d^Jonckheere–Terpstra test was used to assess the trend. HV, hallux valgus; η^2^, partial eta-squared, SS = standardized statistic

**Fig. 2:**
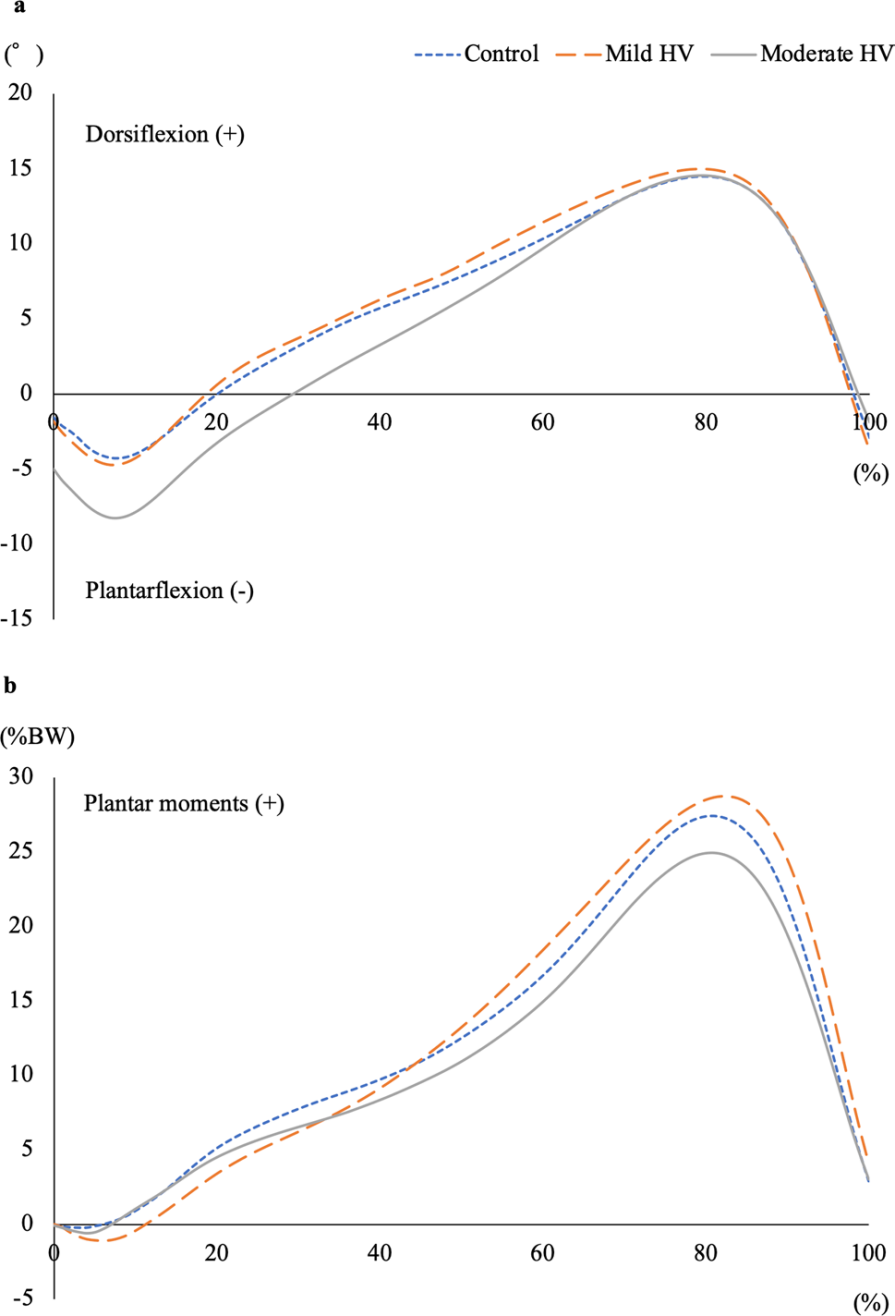
Kinematic data during one gait cycle. Ankle dorsiflexion and plantarflexion angles (**a**) as well as ankle dorsiflexion and plantarflexion moments normalized by body weight (**b**)

Figure 2 shows ankle kinematic data during one gait cycle. The peak ankle plantar flexion angle was not significantly different among the control, mild HV, and moderate HV groups (− 6.1 ± 3.8 versus − 5.5 ± 3.7 versus − 8.8 ± 4.1, *F* = 2.115, *P* = 0.135; Fig. 2a). The peak ankle plantar flexion moment was also not significantly different between the groups (27.7 ± 4.5 versus 28.9 ± 2.5 versus 25.3 ± 2.8, *F* = 2.632, *P* = 0.085; Fig. 2b).

The intra-rater reliability for the US vertical movement of the first TMT joint during the stance phase was considered excellent for the first metatarsal and medial cuneiform locations and good to excellent for the gap between the first metatarsal and medial cuneiform (Table 4).

**Table 4:** Reproducibility of the ultrasonic vertical movement of the first tarsometatarsal joint during the stance phase of gait

	Control		Mild HV		Moderate HV	
	ICC1,3	SEM	ICC1,3	SEM	ICC 1,3	SEM
First metatarsal						
Early stance phase	0.997 (0.995–0.999)	0.234	0.955 (0.879–0.987)	0.259	0.956 (0.860–0.990)	0.701
Middle stance phase	0.995 (0.989–0.998)	0.303	0.957 (0.884–0.987)	0.246	0.944 (0.818–0.988)	0.767
Terminal stance phase	0.993 (0.986–0.997)	0.362	0.973 (0.927–0.992)	0.188	0.942 (0.814–0.987)	0.804
Medial cuneiform						
Early stance phase	0.993 (0.986–0.997)	0.268	0.956 (0.880–0.987)	0.284	0.969 (0.901–0.993)	0.623
Middle stance phase	0.992 (0.984–0.997)	0.466	0.954 (0.877–0.987)	0.301	0.942 (0.812–0.987)	0.876
Terminal stance phase	0.988 (0.976–0.995)	0.563	0.967 (0.911–0.990)	0.257	0.933 (0.784–0.985)	1.007
Gap in the first metatarsal and medial cuneiform						
Early stance phase	0.756 (0.516–0.888)	0.597	0.731 (0.273–0.921)	0.354	0.941 (0.793–0.989)	0.245
Middle stance phase	0.886 (0.773–0.948)	0.375	0.729 (0.267–0.920)	0.334	0.860 (0.509–0.974)	0.443
Terminal stance phase	0.852 (0.707–0.932)	0.463	0.684 (0.148–0.907)	0.400	0.900 (0.677–0.978)	0.420

Values in parentheses are 95% confidence intervals for ICC and lower and upper limits for SEM

HV, hallux valgus; ICC, intraclass correlation coefficient; SEM, standard error of the measurements (calculated using the formula s√1 ICC)

## Discussion

The present study showed in vivo mobility of the first TMT joint in the sagittal plane during the stance phase of gait in young individuals with HV using US synchronized with the MA system. The ICC1,3 was good to excellent, which suggests that the experiment provided a very reliable ultrasonographic evaluation. To the best of our knowledge, this is the first study to reveal that the dynamic hypermobility of the first TMT joint, especially in the medial cuneiform, correlates with the severity of the HV deformity in young individuals. Furthermore, the Jonckheere–Terpstra test showed greater plantar displacement of the medial cuneiform with higher HV severity across all gait phases. Although no significant differences in gait parameters, peak ankle plantarflexion angle, or peak ankle plantarflexion moment were observed between the control and HV groups, the features of the first TMT joint mobility during the stance phase could be detected in HV feet via the US/MA system.

The three-dimensional mobility of the first ray, including the talonavicular, cuneonavicular, and first TMT joints, was also recently assessed through weight-bearing computed tomography imaging [8, 25]. The first metatarsal showed, on average, 2.0° ± 1.3° of dorsiflexion, 2.6° ± 1.4° of inversion, and 1.1° ± 0.7° of adduction relative to the medial cuneiform under loading in middle-aged healthy volunteers [8]. This static 3D mobility was significantly greater in HV feet than in healthy feet. In the present study, a stable medial cuneiform was identified during the entire phase of gait in the control group, which could be considered normal dynamics of the first TMT joint. The mild HV group showed a significant plantar shift of the medial cuneiform during the early stance phase of gait compared with the control group; this shift was also found in the middle and terminal stance phases in the moderate HV group compared with the control group.

Plantar displacement of the medial cuneiform in the moderate HV group may indicate laxity of each of the naviculocuneiform, intercuneiform 1–2, and first TMT joints. A cadaveric study revealed that the naviculocuneiform, first TMT, and talonavicular joints accounted for 50%, 41%, and 9% of the total range of motion in the sagittal plane of the first ray, respectively [26]. In a 3D computed tomography analysis during weight-bearing, the mobility of the intercuneiform 1–2 joint was greater in HV feet than in healthy feet. Thereafter, a significant increase was found in dorsiflexion and inversion displacement of the intermediate cuneiform relative to the medial cuneiform [27]. Hypermobility of the joints formed between the medial cuneiform and surrounding bones, such as the naviculocuneiform, intercuneiform 1–2, and first TMT joints, may occur depending on HV severity and lead to progression of HV. Similarly validating this observation, plantar displacement of the medial cuneiform was associated with HV severity in this study. It was significantly greater in the mild HV group in the early stance phase and in the moderate HV group in the middle and terminal stance phases compared to that in the control group. Historically, hypermobility of the first ray has been reported to contribute to reduction of the medial longitudinal arch, which may explain our results [28]. Additionally, as body weight shifts forward from the middle to the terminal stance phase, midfoot eversion occurs [9]. The increased pressure at this moment could contribute to a tendency toward plantar displacement of the medial cuneiform associated with the HV severity. Further prospective studies are warranted to clarify the relationship between HV development and the mobility of the naviculocuneiform and intercuneiform 1–2 joints. A dynamic evaluation using the US/MA system has the potential to detect characteristic dynamics of the medial cuneiform during gait that cannot be evaluated with previous techniques. With regards to surgical management of HV, preoperative findings of the medial cuneiform dynamics during gait may help determine indications for additional procedures targeting the medial cuneiform, which may serve to prevent postoperative recurrence of HV.

This study had several limitations. First, the gait analysis was performed under an uncontrolled gait speed, which might have affected the mobility of the first TMT joint [29]. However, a comfortable gait speed is believed to be suitable for evaluation because it is most frequently used in daily life. Second, the mobility of the naviculocuneiform joint was not assessed. A future evaluation of the mobility of the naviculocuneiform joint using the US/MA system is required to quantify the hypermobility of the first ray during gait in HV feet. Third, considering these results were analyzed only for young adults, further investigation should be conducted to explore the dynamics of the first TMT joint during gait in middle-aged and older adults, the age at which HV is more likely to occur. Fourth, we cannot exclude the possibility that the US probe may have affected ankle kinematics during gait. Finally, in this study, the probe orientation was not identified because measurements were not taken with reference to a global reference frame.

## Conclusions

Quantification of first TMT joint mobility in the sagittal plane using the US/MA system revealed the detailed biomechanical mobility of the first TMT joint. Significantly greater mobility was found depending on the severity of the HV deformity. Our results provide a novel evaluation method for early detection of first TMT joint hypermobility, especially in the medial cuneiform, and may aid in predicting the progression and postoperative recurrence of HV deformity in daily clinical practice in the future. Furthermore, our findings indicate that hypermobility of the first TMT joint can already be present in young HV cases, suggesting the significance of preventing HV deformity from an early age.

### Supplementary Information

Below is the link to the electronic supplementary material.

Supplementary file1 (DOCX 7436 KB)

## Data Availability

The datasets generated during and/or analyzed during the current study are available from the corresponding author on reasonable request.
