# Supplementary material for: First tarsometatarsal joint mobility in hallux valgus during gait: A synchronized ultrasound and three-dimensional motion capture analysis
Source: J Med Ultrason (2001). 2024 Mar 28;51(2):331–9. doi: 10.1007/s10396-024-01414-2 (PMC11098882; doi:10.1007/s10396-024-01414-2)
Supplement: Supplementary file 1 — Supplementary file1 (DOCX 7436 KB) [file 10396_2024_1414_MOESM1_ESM.docx]

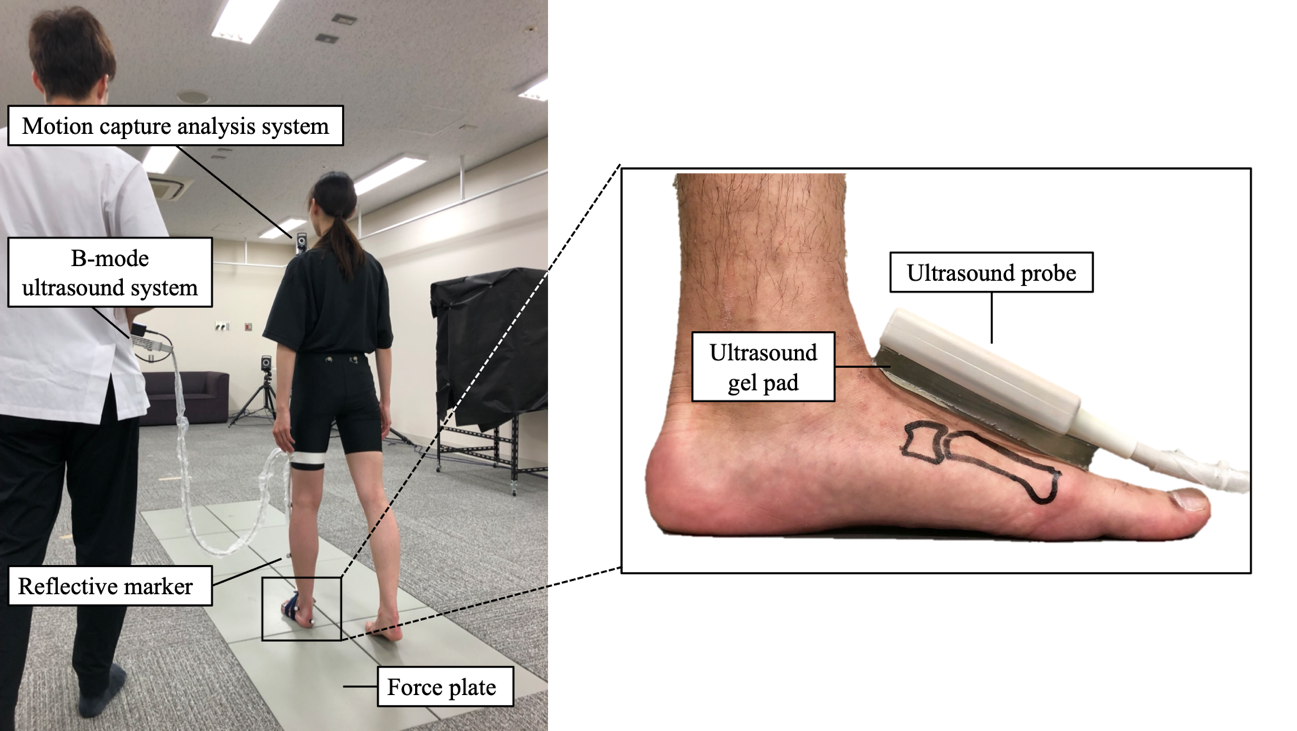
**Supplement Figure 1**. Gait analysis using the synchronized ultrasound and a three-dimensional motion analysis (US/MA) system. The ultrasound probe was attached just above the TMT joint via an ultrasound gel pad.

**
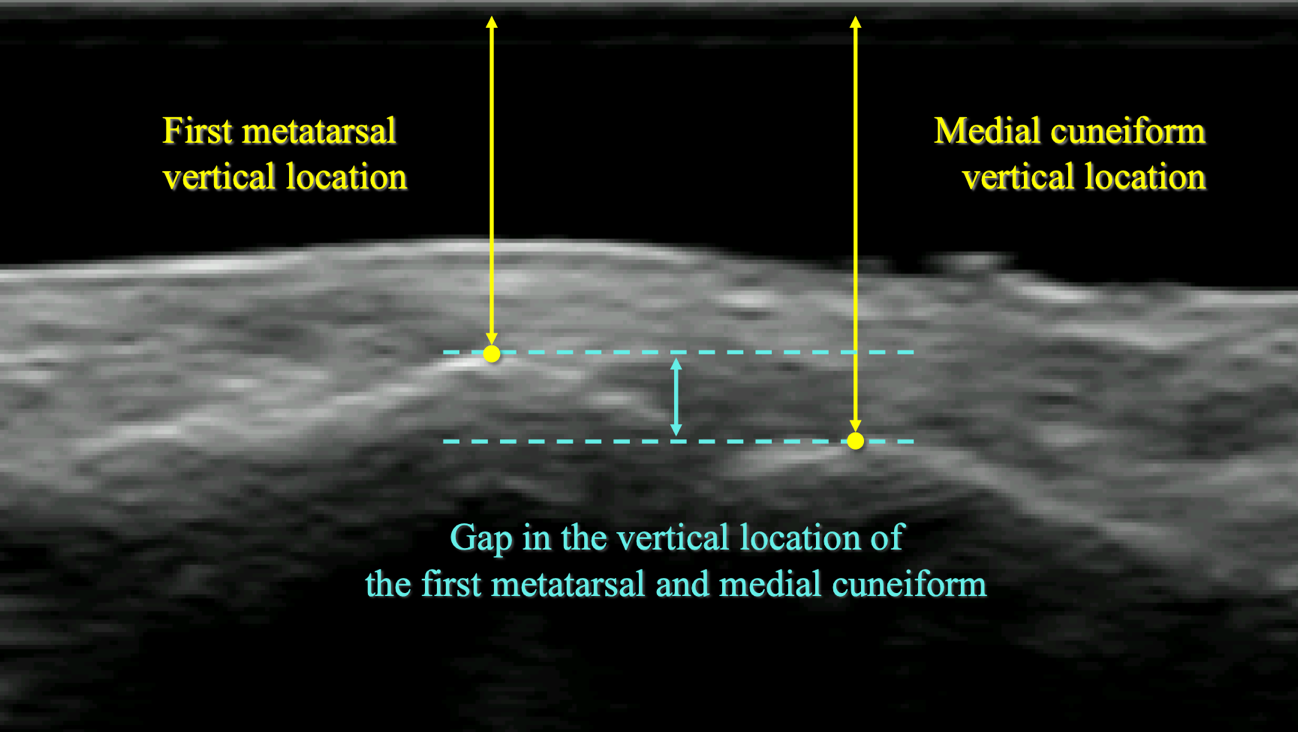
Supplement Figure 2**. Ultrasound video screen shows the first metatarsal and medial cuneiform. The vertical locations of the first metatarsal and medial cuneiform were defined as the vertical distance from the top of the screen to the dorsum of each bone.
